# Th2/Th1 Cytokine Imbalance Is Associated With Higher COVID-19 Risk Mortality

**DOI:** 10.3389/fgene.2021.706902

**Published:** 2021-07-16

**Authors:** Ana B. Pavel, Jacob W. Glickman, James R. Michels, Seunghee Kim-Schulze, Rachel L. Miller, Emma Guttman-Yassky

**Affiliations:** ^1^Computational Biology Lab, Department of Biomedical Engineering, University of Mississippi, University, MS, United States; ^2^Laboratory of Inflammatory Skin Diseases, Department of Dermatology, Icahn School of Medicine at Mount Sinai, New York, NY, United States; ^3^Human Immune Monitoring Center, Icahn School of Medicine at Mount Sinai, New York, NY, United States; ^4^Division of Clinical Immunology, Department of Medicine, Icahn School of Medicine at Mount Sinai, New York, NY, United States

**Keywords:** COVID-19, SARS-CoV-2, T-cells, Th1, Th2, Th17, gene expression, serum proteomics

## Abstract

A major component of COVID-19 severe respiratory syndrome is the patient’s immune response to the SARS-CoV-2 virus and the consequential multi-organ inflammatory response. Several studies suggested a potential role of CD4^+^ T cells in COVID-19 severe respiratory syndrome. We first hypothesized that there is a type 2 helper (Th2)/type 1 helper (Th1) imbalance in older age, male, asthma, smokers, and high ACE2 expression phenotype in the airway of non-infected patients. Next, we hypothesized that a Th2/Th1 imbalance may predict higher mortality in COVID-19 infected hospitalized patients with and without patient reported current asthma. We first analyzed publicly available gene expression from the sputum of 118 moderate-to-severe asthma patients and 21 healthy controls, and from nasal epithelium of 26 healthy current smokers and 21 healthy never smokers. Secondly, we profiled 288 new serum proteomics samples measured at admission from patients hospitalized within the Mount Sinai Health System with positive SARS-CoV-2 infection. We first computed Th1 and Th2 pathway enrichment scores by gene set variation analysis and then compared the differences in Th2 and Th1 pathway scores between patients that died compared to those that survived, by linear regression. The level of Th2/Th1 imbalance, as determined by the enrichment score, was associated with age, sex, and ACE2 expression in sputum, and with active smoking status in nasal epithelium (*p* < 0.05). Th2/Th1 imbalance at hospital admission in sera of patients was not significantly associated with death from COVID-19 (*p* = 0.11), unless evaluated in the asthmatic strata (*p* = 0.01). Using a similar approach we also observed a higher Th17/Th1 cytokine imbalance in all deceased patients compared to those that survived (*p* < 0.001), as well as in the asthmatic strata only (*p* < 0.01). Th2/Th1 imbalance is higher in the sera of asthma patients at admission that do not survive COVID-19, suggesting that the Th2/Th1 interplay may affect patient outcomes in SARS-CoV2 infection. In addition, we report that Th17/Th1 imbalance is increased in all patients that die of COVID-19.

## Introduction

SARS-CoV-2 is a highly infectious pathogen that is quickly spreading across the world with a mortality rate currently estimated to be between 2 and 10% in hospitalized patients (Deng et al., 2020; Garg et al., 2020). Epidemiological studies have identified several comorbidities associated with higher risk of hospitalizations and mortality due to SARS-CoV-2 infection (Chen T. et al., 2020; Garg et al., 2020). Patients with pre-existing conditions such as asthma, hypertension, smoking, and old age are especially at high risk of succumbing to this viral infection (Chen T. et al., 2020; Garg et al., 2020; Pavel et al., 2021). Genomic and proteomic profiles are currently being evaluated to understand further the molecular mechanisms triggered by SARS-CoV-2 infection.

A major component of COVID-19 severe respiratory syndrome is the patient’s immune system response to the pathogen, and the consequential multi-organ inflammatory response. Several studies suggested a potential role of circulating CD4^+^ T cells in COVID-19 severe respiratory syndrome and multi-organ systemic inflammation (Chen and John Wherry, 2020; Wu and Yang, 2020). CD4^+^ T cells specific for the SARS-CoV-2 spike protein have been identified in acute infection, harboring a T helper (Th)1 cell cytokine profile (Weiskopf et al., 2020). Another cross-sectional study describes a lower proportion of IFNγ-producing type 1 helper (Th1) cells in sera from patients with severe disease (Chen G. et al., 2020). However, the comprehensive mechanisms of Th2 immunity have not yet been evaluated in subsequent COVID-19 clinical outcomes. One limited cytokine study suggested that SARS-CoV-2 infection initiates increased secretion of plasma Th2 cytokines such as IL-4, IL-13, and IL-10 (Huang et al., 2020), unlike other SARS-CoV infections (Wong et al., 2004). Hence, the role of Th2 cell-type responses, and its relationship with Th1 responses, especially as it relates to affecting COVID-19 disease outcome, remains unclear (Weiskopf et al., 2020). Examining the interplay of major immune axes in COVID-19 patients, and particularly in those with a Th2 specific underlying condition such as asthma, may shed light on the role of Th2/Th1 imbalance in predicting COVID-19 disease outcome.

In this study we aim to evaluate the interplay between Th1 and type 2 helper (Th2) cells cytokines in populations with a high risk of COVID-19 severe symptoms such as older individuals, males, patients with high ACE2 expression by sputum gene expression profiling (Kuo et al., 2017), in smokers by nasal epithelium gene expression profiling (Zhang et al., 2010), and in patients that died of COVID-19 by serum proteomics profiling. Due to the Th2 role in suppressing inflammation and inhibiting antiviral Th1 responses, a shift in the Th1 to Th2 cytokine balance can result in chronic infections, such as pulmonary infections (Herring et al., 2004; Lee and Ashkar, 2018) and hepatitis B (Saxena and Kaur, 2015). Furthermore, Th2 versus Th1 increases have been previously reported in asthma and other allergic diseases (Kearley et al., 2007; Kuo et al., 2017). However, the Th2/Th1 interplay in asthma patients with a severe viral immune response such as SARS-CoV-2 syndrome, have not yet been scrutinized. We hypothesized that the interplay between Th1 and Th2 pathways, as defined by the difference in protein enrichment scores in sera, plays a role in COVID-19 severe disease outcome and may predict greater mortality following COVID-19 infection, particularly in asthma patients. Previous studies also have associated Th17 with asthma and immune responses to pathogens (Wu and Yang, 2020); thus, we also evaluated whether Th17/Th1 imbalances may predict worse outcomes following COVID-19 infection.

## Materials and Methods

### Patient Cohorts

We first analyzed publicly available gene expression data (GSE76262) (Green et al., 2002; Kuo et al., 2017) from the sputum of 118 moderate-to-severe asthma patients (mean age ± SD 51 ± 13.6) and 21 healthy controls (mean age ± SD 37.8 ± 13.6), and from nasal epithelium (GSE16008) (Zhang et al., 2010) of 26 healthy current smokers (mean age ± SD 41.7 ± 10.4), and 21 healthy never smokers (36.5 ± 11.6).

Next, we evaluated 288 new serum samples from hospitalized patients (mean age 64 ± 15; Table 1) with a positive SARS-CoV-2 polymerase chain reaction (PCR) test in the Mount Sinai Health System from 03/01/20 to 06/07/20.

**TABLE 1:** Demographics of hospitalized COVID-19 patients.

	Asthma			No asthma		
	Deceased, *n* = 5	Survived, *n* = 16	*p*	Deceased, *n* = 43	Survived, *n* = 224	*p*
Age (mean ± SD)	59.2 ± 13.4	62 ± 10.6	0.58	70.95 ± 14.6	62.7 ± 15.1	0.001
Sex	1M/4F	5M/11F	1	24M/19F	145M/79F	0.3
Race/ethnicity	3B/1H/1W/0A	3B/8H/1W/0A	0.2	11B/15H/7W/3A	59B/56H/45W/13A	0.7
Smoking status	1C/2N/2F/0UNK	3C/7N/5F/1UNK	1	3C/15N/10F/15UNK	9C/104N/55F/56UNK	0.3
Obesity	3Yes/2No	9Yes/7No	1	11Yes/32No	74Yes/150No	0.4
Diabetes	2Yes/3No	6Yes/10No	1	14Yes/29No	47Yes/177No	0.1
COPD	2Yes/3No	1Yes/15No	0.1	3Yes/40No	11Yes/213No	0.5

*SD, standard deviation; smoking status: C, current smoker; N, never smoker; F, former smoker; UNK, unknown smoking status, by patient report; obesity: Yes, BMI ≥ 30; No, BMI < 30.*

### Serum Profiling

Serum cytokines were profiled by Proseek Multiplex OLINK Proteomics as previously described (Brunner et al., 2017, 2019; He et al., 2020). First, serum samples were collected, centrifuged, and stored at −80 °C until further processing. Aliquots were analyzed with an OLINK Proseek^®^ multiplex assay (Lind et al., 2015; Soderlund et al., 2016) a proximity extension assay (PEA) technology with oligonucleotide-labeled antibody probe pairs that bind to their respective targets (Assarsson et al., 2014). Upon binding of antibody pairs to their respective targets, DNA reporter molecules bound to the antibodies gave rise to new DNA amplicons with each ID-barcoding their respective antigens. The amplicons were subsequently quantified using a Fluidigm BioMark^TM^ HD real-time PCR platform (Lind et al., 2015). Serum was analyzed using Inflammation I panel (Lind et al., 2015). OLINK data by subject are available on Gene Expression Omnibus (GSE178399).

### Data Analysis

We computed enrichment scores for Th1 and Th2 immune pathways (Glickman et al., 2020) by gene set variation analysis (Hanzelmann et al., 2013), and modeled the Th2/Th1 balance as the difference between Th2 and Th1 enrichment scores.

Using a linear regression model, we compared the Th2/Th1 gene enrichment score in the airway between younger and older age groups, males and females, asthma and controls, and healthy active smokers and non-smokers.

We next compared the levels in Th2/Th1 protein enrichment score in serum between patients that subsequently died of COVID-19 compared to those that survived, adjusting by age that was the only significant covariate associated with mortality in our COVID-19 cohort (Table 1). We then stratified by the current asthma status as reported upon hospital admission. The Th2 pathway includes immune markers such as IL-4, IL-13, CCL7, TSLP, CCL13, CCL11, IL10, IL33, and IL-5, while Th1 includes CXCL9, CXCL10, CXCL11, IFNG, IL-2, IL-8, CCL3, and IL-12B (Table 2).

**TABLE 2:** Proteins profiled in COVID-19 serum by pathway.

Th1 pathway	IFNG, CXCL9, CXCL10, CXCL11, IL-2, IL-8, CCL3, IL-12B
Th2 pathway	IL-4, IL-13, CCL7, TSLP, CCL13, CCL11, IL10, IL33, IL-5
Th17 pathway	IL-6, IL17A, CXCL1, IL12B, S100A12, CCL20

Using a similar approach, we also evaluated Th17/Th1 imbalance in sera between patients that died of COVID-19 compared to those that survived, and also stratified by the current asthma status. The Th17 pathway includes markers such as IL-6, IL17A, CXCL1, IL12B, S100A12, and CCL20 (Table 2).

## Results

**FIGURE 1:**
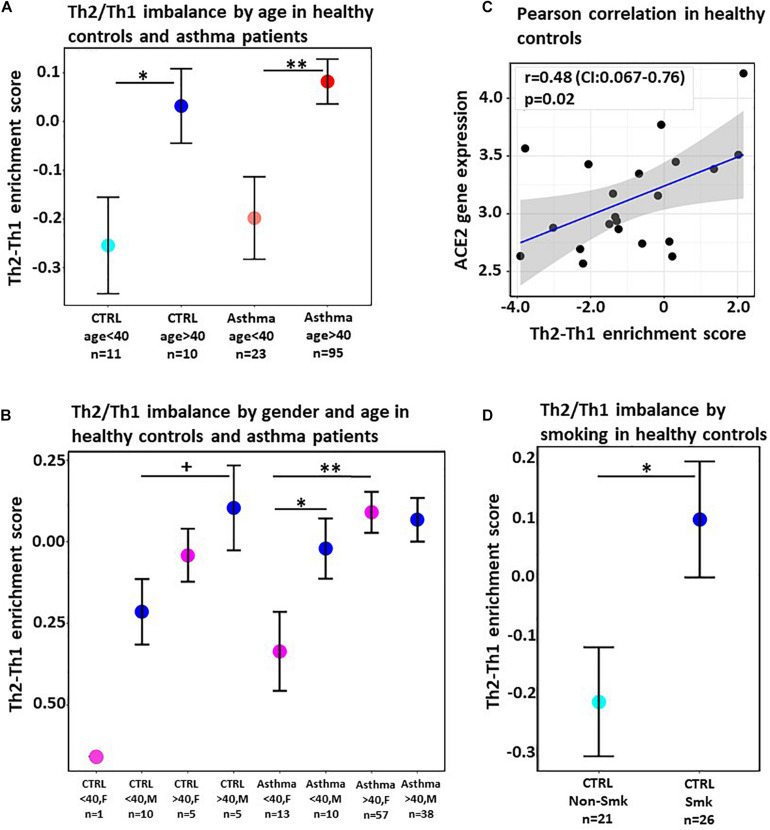
**(A)** Mean difference in Th2 and Th1 enrichment scores in the sputum of healthy individuals (CTRL) and severe asthma patients stratified by age. Horizontal bars denote standard error. **(B)** Mean difference in Th2 and Th1 enrichment scores in sputum of healthy individuals (CTRL) and of asthma patients in both males (M) and females (F) stratified by age. Horizontal bars denote standard error. **(C)** Correlation scatterplot between Th2/Th1 enrichment score and *ACE2* gene expression in healthy individuals. Gray shaded area denotes the 95% confidence interval. Pearson correlation coefficient and *p*-value are provided. **(D)** Mean difference in Th2 and Th1 enrichment scores in nasal epithelium of healthy smokers and healthy never smokers. Horizontal bars denote standard error. +*p* < 0.1, *p < 0.05, **p < 0.01.

The Th2/Th1 cytokine imbalance was significantly increased in patients with ages greater than 40 years in both asthma (*p* < 0.01) and healthy control (*p* < 0.05) groups (Figure 1A), suggesting that the high Th2/Th1 enrichment score in sputum is associated with age across the entire patient cohort.

We further evaluated the association of the Th2/Th1 cytokine imbalance with sex (Figure 1B) and found an increasing trend in males compared to females in healthy subjects, as well as a significant higher enrichment score in younger male patients with asthma (*p* < 0.05), congruent with the currently known COVID-19 risk populations.

Given the role of ACE2 on SARS-CoV-2 entry and replication (Zhang et al., 2020), we also evaluated the correlation between ACE2 expression as a known risk for COVID-19 severe disease and the Th2/Th1 signature score. Using Pearson’s correlation, we found a significant association between ACE2 and the Th2/Th1 imbalance (*r* = 0.44, *p* < 0.05) in sputum from healthy individuals (Figure 1C). There was no association between Th2/Th1 enrichment score and ACE2 expression in sputum of asthma patients (*p* = 0.58) (Kuo et al., 2017). The lack of association between Th2/Th1 shift and ACE2 expression in asthma patients may be due to a more reduced ACE2 phenotype previously reported in asthma (Branco et al., 2020; Yao et al., 2020).

Furthermore, we evaluated smoking status, another known risk factor for COVID-19 severe disease, in nasal epithelium from a cohort of 26 healthy current smokers and 21 healthy never smokers, with no statistical differences in age and gender between current and never smokers (GSE16008) (Zhang et al., 2010). The Th2/Th1 imbalance was significantly higher in current smokers compared to never smokers (*p* < 0.05; Figure 1D).

**FIGURE 2:**
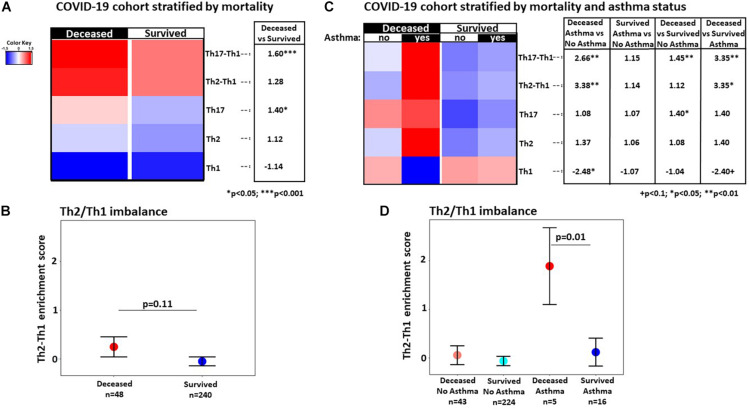
**(A)** Heatmap of mean enrichment score by gene set variation analysis in all patients in the hospitalized COVID-19 cohort, stratified by subsequent mortality, with fold-change differences provided in the side table. **(B)** Mean difference in Th2 and Th1 enrichment scores in deceased versus survived patients. **(C)** Heatmap of mean enrichment scores by gene set variation analysis in asthma and non-asthma patients, stratified by mortality, with fold-changes provided in the side table. **(D)** Mean difference in Th2 and Th1 enrichment scores in deceased versus survived patients stratified by asthma status; means were estimated by a linear regression with age adjustment; ^+^*p* < 0.1, *p < 0.05, **p < 0.01, ***p < 0.001.

Furthermore, we evaluated major immune axes such as Th1, Th2, and Th17, as well as the Th2/Th1 and Th17/Th1 cytokine imbalance in serum proteomics data profiled from a new cohort of 288 COVID-19 infected patients at admission (Figure 2). While the Th2/Th1 cytokine imbalance at admission was not significantly increased (*p* = 0.11; Figures 2A,B) in patients who died compared to those that survived, the imbalance became significant when examining the strata of patients with asthma (*p* = 0.01; Figures 2C,D). While the Th2 pathway showed a non-significant increase in asthma patients that died compared to those that survived (*p* = 0.45; Figure 2C), the Th1 pathway showed a non-significant decrease (*p* = 0.07; Figure 2C), resulting in a significant Th2/Th1 imbalance (*p* = 0.01; Figures 2C,D). While we also observed a modest increase in the Th17 pathway that achieved significance only across all patients that died compared to those that survived (*p* = 0.01; Figure 2A), the Th17/Th1 imbalance was significantly increased in all patients that died compared to those that survived (*p* = 0.0002; Figure 2A), as well as in the asthma subset only (*p* = 0.003; Figure 2C).

## Discussion

To our knowledge, we demonstrate for the first time that Th2/Th1 cytokine imbalance in the airway is associated with major COVID-19 risk factors such as age, sex, high ACE2 expression phenotype, and smoking, in the airway of non-infected patients by data mining of multiple gene expression studies. Importantly, these data suggest that Th2 inhibition (Barranco et al., 2017) might potentially offer protection against COVID-19 severe symptoms, provided that there are minimal effects on Th1. Our findings may help guide personalized medicine decisions, particularly in patients that are eligible to receive Th2 inhibitors such as those with asthma or other atopic conditions (Barranco et al., 2017; Grey and Katelaris, 2019; Guttman-Yassky et al., 2019). However, this can truly be validated through clinical trials and longitudinal studies.

In a cohort of patients admitted with COVID-19, we further demonstrate that Th2/Th1 imbalance at admission is significant in asthma patients that subsequently died of COVID-19 compared to those that survived. Our findings provide new insights on how to optimize COVID-19 treatments and prevention strategies in specific cohorts of patients, such as patients with asthma and other atopic conditions that present with Th2/Th1 imbalance.

Our results suggest the possible positive role of Th1 cytokine production in subsequent COVID-19 disease outcomes, particularly in the setting of asthma, as previously reported in other viral infections (Herring et al., 2004; Saxena and Kaur, 2015; Lee and Ashkar, 2018). The Th2/Th1 imbalance observed in asthma patients that died of COVID-19 suggests that the Th2 axis may be harboring Th1 immunity. Our approach suggests the importance of Th1 activity in fighting COVID-19 infection, particularly in asthma patients.

Our data also suggest a possible association between worse outcomes and higher Th17 in all patients. This is further supported by a recent report showing that IL-17 antagonism lowers ACE2 (Pavel et al., 2021) (a receptor critically involved in SARS-CoV-2 cell entry) expression in psoriasis patients (Krueger et al., 2019), and that higher Th17 response has been associated with worse allergic asthma severity (Shi et al., 2011).

We acknowledge the small sample size of analyzed patient subgroups, the lack of immunological profiles prior infection in our serum COVID-19 cohort, and missing smoking status in approximately 25% of non-asthma patients, as limitations. Nevertheless, our data show evidence that the Th2/Th1 and Th17/Th1 imbalances at hospital admission may predict COVID-19 disease outcome in asthma patients.

Future work will evaluate the Th2/Th1 score in larger clinical studies in patients with asthma and other atopic conditions. More complete toxicology profiles will be evaluated, as well as the potential of developing a predictor for COVID-19 mortality risk in asthma patients based on Th2/Th1 score.

In summary, while additional studies are needed to uncover the mechanistic basis of varying COVID-19 clinical presentations, our data associate the Th2/Th1 and Th17/Th1 imbalance in cytokine expression with COVID-19 mortality, suggesting that clinical trials of Th2 (Barranco et al., 2017) and/or Th17 specific inhibition may reveal potentially protective mechanisms against deleterious viral effects by enhancing Th1 immunity.

## Data Availability Statement

The datasets presented in this study can be found in online repositories. The OLINK serum proteomics data can be found on Gene Expression Omnibus (GSE178399).

## Ethics Statement

The studies involving human participants were reviewed and approved by the Department of Medicine at the Icahn School of Medicine; approval number: IRB-20-03788. The patients/participants provided their written informed consent to participate in this study.

## Author Contributions

AP designed the study, conducted the data analysis and interpretation, and wrote the manuscript. JG contributed to the data analysis and interpretation and reviewed the manuscript. JM helped with data check and the revision of the manuscript. SK-S contributed to the OLINK proteomics profiling. RM and EG-Y proposed the clinical protocol and contributed to the study design, data interpretation and writing of the manuscript. All authors contributed to the article and approved the submitted version.

## Conflict of Interest

AP is an employee of the University of Mississippi and has received a research grant from Mount Sinai. EG-Y is an employee of Mount Sinai and has received research funds (grants paid to the institution) from: Abbvie, Celgene, Eli Lilly, Janssen, Medimmune/Astra Zeneca, Novartis, Pfizer, Regeneron, Vitae, Glenmark, Galderma, Asana, Innovaderm, Dermira, and UCB. EG-Y is also a consultant for Sanofi Aventis, Regeneron, Stiefel/GlaxoSmithKline, MedImmune, Celgene, Anacor, AnaptysBio, Dermira, Galderma, Glenmark, Novartis, Pfizer, Vitae, Leo Pharma, Abbvie, Eli Lilly, Kyowa, Mitsubishi Tanabe, Asana Biosciences, and Promius. JG, SK-S and RM are employees of Mount Sinai and have no other financial relationships to disclose. JM has no financial relationships to disclose. The reviewer RJ declared a past co-authorship with the authors AP, JG, and EG-Y to the handling editor.

## Abbreviations

ACE2: angiotensin converting enzyme 2
COVID-19: coronavirus disease 2019
*p*: *p*-value
PCR: polymerase chain reaction
SARS-CoV-2: severe acute respiratory syndrome coronavirus 2
Th1: type 1 helper cells
Th17: type 17 helper cells
Th2: type 2 helper cells.
